# Cryo-electron Microscopy Study of the Genome Release of the Dicistrovirus Israeli Acute Bee Paralysis Virus

**DOI:** 10.1128/JVI.02060-16

**Published:** 2017-01-31

**Authors:** Edukondalu Mullapudi, Tibor Füzik, Antonín Přidal, Pavel Plevka

**Affiliations:** a Structural Virology, Central European Institute of Technology, Masaryk University, Brno, Czech Republic; b Department of Zoology, Fishery, Hydrobiology, and Apidology, Faculty of Agronomy, Mendel University in Brno, Brno, Czech Republic; Instituto de Biotecnologia/UNAM

**Keywords:** virus, Apis mellifera, honey bee, honeybee, Picornavirales, Dicistroviridae, Aparavirus, virion, structure, cryo, electron microscopy, capsid, genome, release, uncoating, colony collapse disorder, CCD, empty

## Abstract

Viruses of the family Dicistroviridae can cause substantial economic damage by infecting agriculturally important insects. Israeli acute bee paralysis virus (IAPV) causes honeybee colony collapse disorder in the United States. High-resolution molecular details of the genome delivery mechanism of dicistroviruses are unknown. Here we present a cryo-electron microscopy analysis of IAPV virions induced to release their genomes *in vitro*. We determined structures of full IAPV virions primed to release their genomes to a resolution of 3.3 Å and of empty capsids to a resolution of 3.9 Å. We show that IAPV does not form expanded A particles before genome release as in the case of related enteroviruses of the family Picornaviridae. The structural changes observed in the empty IAPV particles include detachment of the VP4 minor capsid proteins from the inner face of the capsid and partial loss of the structure of the N-terminal arms of the VP2 capsid proteins. Unlike the case for many picornaviruses, the empty particles of IAPV are not expanded relative to the native virions and do not contain pores in their capsids that might serve as channels for genome release. Therefore, rearrangement of a unique region of the capsid is probably required for IAPV genome release.

**IMPORTANCE** Honeybee populations in Europe and North America are declining due to pressure from pathogens, including viruses. Israeli acute bee paralysis virus (IAPV), a member of the family Dicistroviridae, causes honeybee colony collapse disorder in the United States. The delivery of virus genomes into host cells is necessary for the initiation of infection. Here we present a structural cryo-electron microscopy analysis of IAPV particles induced to release their genomes. We show that genome release is not preceded by an expansion of IAPV virions as in the case of related picornaviruses that infect vertebrates. Furthermore, minor capsid proteins detach from the capsid upon genome release. The genome leaves behind empty particles that have compact protein shells.

## INTRODUCTION

The productivity of many flowering food plants depends on the pollination provided by the western honeybee (Apis mellifera) (1). Honeybees are also critical for maintaining the biodiversity of wild flowering plants (2). Winter honeybee colony mortality has been increasing in North America and Europe over the last couple of decades, leading to a decline in the number of honeybee colonies (3–5). Virus infections are a major factor in the winter honeybee colony losses (6, 7). Generally, honeybee viruses cause latent asymptomatic infections; however, they can occasionally lead to outbreaks characterized by high virus titers. For some of these viruses, such outbreaks are connected with increased virulence, resulting in the deaths of individual workers as well as of whole colonies (6). Israeli acute bee paralysis virus (IAPV) is a member of the Aparavirus genus of the family Dicistroviridae (8–10). IAPV, Kashmir bee virus, and acute bee paralysis virus constitute a cluster of closely related viruses that are distributed worldwide (11). The spread of these viruses is accelerated by transmission by the parasitic mite Varroa destructor (6, 11–13). IAPV has been linked to colony collapse disorder (CCD), still a largely unexplained rapid loss of adult bees from colonies in the United States (14–17), while acute bee paralysis virus has been associated with a similar rapid adult bee mortality in Europe (11, 18). Other dicistroviruses are pathogens of economically important arthropods, including crickets and shrimps (19).

Viruses of the family Dicistroviridae have nonenveloped icosahedral capsids protecting linear, single-stranded, positive-sense RNA genomes of 8,500 to 10,200 nucleotides (20). The genomes of dicistroviruses include two nonoverlapping open reading frames, ORF1 and ORF2, which encode polyproteins containing nonstructural and structural (capsid-forming) proteins, respectively. The polyproteins are cotranslationally and posttranslationally cleaved by viral proteases to produce functional subunits. The major capsid proteins VP1 to VP3 of IAPV form the capsid shell with pseudo-T=3 icosahedral symmetry, whereas VP4 is a small protein attached to the inner surface of the capsid (21). The major capsid proteins of IAPV have a jelly roll β-sandwich fold common to many other virus capsid proteins (21). The maturation of the capsids of viruses of the order Picornavirales depends on the cleavage of the capsid protein VP4 from the N terminus of a precursor subunit, VP0. In dicistroviruses, the precursor cleavage generates VP4 and VP3 subunits (19, 22). It was proposed previously that a conserved Asp-Asp-Phe (DDF) motif, which is a part of the VP1 subunit that is exposed inwards toward the virion cavity, is involved in VP0 cleavage (19, 22, 23).

In order to initiate infection, virus genomes need to be released from capsids and transferred across the biological membrane into the cell cytoplasm. Previously, the genome release of another dicistrovirus, triatoma virus (TrV), was analyzed by cryo-electron microscopy (cryo-EM) of full and empty TrV particles to resolutions of 15 to 22 Å (24). TrV RNA release led to empty capsids that had a size similar to that of the native virions. A tectonic model of subunit movements was described, according to which the individual capsid proteins rotated within the capsid (24). Subsequently, the empty capsids of TrV disassembled into small, symmetrical, lip-shaped particles that were probably dimers of pentamers of capsid protein protomers (21, 24). It was proposed that capsid cracking or dismantling was associated with the RNA externalization process of TrV (24).

Genome release and delivery have been studied more extensively for viruses of the family Picornaviridae, and for enteroviruses in particular (25–30). The genome release of enteroviruses is preceded by structural changes of the capsid leading to the formation of an expanded A particle that is induced by receptor binding or by the low pH of late endosomes (25, 31–33). The A particles of enteroviruses contain pores at icosahedral 2-fold symmetry axes (26–31, 34–36) that were speculated to allow the release of the genome and of VP4 subunits. Another type of pores, located between 2-fold and 5-fold symmetry axes, serve for the externalization of N-terminal arms of VP1 subunits (28, 34).

Here we present a high-resolution cryo-EM analysis of IAPV virions induced to release their genomes *in vitro*. We show that IAPV genome release is not preceded by the formation of A particles. Furthermore, IAPV empty capsids are compact and do not contain pores that might serve as channels for genome release.

## RESULTS AND DISCUSSION

### *In vitro* induction of IAPV genome release.

**FIG 1:**
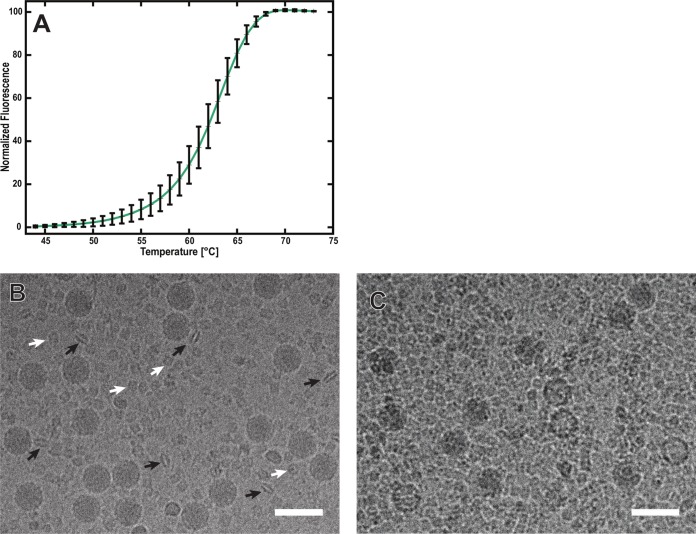
Increased temperature triggers genome release of IAPV. (A) Thermal stability of IAPV virions. IAPV virions were mixed with Sybr green II dye and heated to the indicated temperatures (*x* axis). The fluorescence signal increased as the dye bound to RNA. Error bars indicate standard deviations of the means (*n* = 3). See Materials and Methods for details. Cryo-electron micrographs show native IAPV virions at room temperature (B) and empty IAPV particles (C), induced by incubating the virus at 63°C for 10 min. The black arrows in panel B indicate “lip-shaped” particles formed by IAPV capsid proteins. The white arrows indicate complexes of the honeybee protein hexamerin that contaminated the virus purification. Bars, 50 nm.

The mechanism of dicistrovirus genome release *in vivo* has not been studied. However, the genome release of related picornaviruses is induced by receptor binding or by the low pH in endosomes (25, 31–33, 37). Furthermore, it was shown that elevated temperatures trigger conformational changes to the capsids of many picornaviruses that lead to genome release *in vitro* (29). Therefore, we used an RNA-binding fluorescent-dye assay to measure the stability of IAPV virions at increasing temperatures (Fig. 1A). The exposure of IAPV to 63°C allowed 50% of the RNA genome to interact with the fluorescent dye (Fig. 1A). It is possible that the increased temperature induced the release of the RNA genome from IAPV virions or that the RNA-binding fluorescent dye could enter the virus particles. Furthermore, cryo-EM images show that IAPV virions heated to 63°C for 10 min contained 8% empty particles (Fig. 1B and C). A similar temperature-induced genome release was described previously for TrV (24). The temperature required to induce the release of the IAPV genome is higher than those required for the genome release of vertebrate picornaviruses, which are in the range of 42 to 56°C (29). This higher stability might be an adaptation of IAPV virions to remain intact in plant nectar with high ionic strength (38).

### Comparison of structures of native and heated IAPV virions.

**FIG 2:**
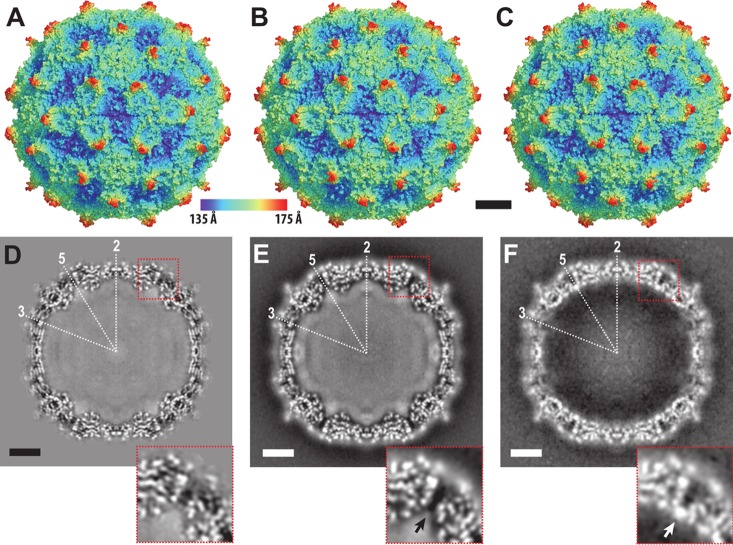
Structures of native IAPV virions and full and empty particles heated to 63°C. An X-ray structure of a native virion of IAPV (A) and cryo-EM structures of full virions heated to 63°C (B) and of empty particles prepared by heating virions to 63°C (C) are shown. The solvent-accessible surfaces are rainbow colored based on their distances from the particle center. Central slices of electron density maps of native virions (D), full virions heated to 63°C (E), and empty particles heated to 63°C (F) are shown in the bottom row. White areas indicate areas with high electron density values. The positions of selected icosahedral symmetry axes are labeled. The insets in panels D to F show details of electron density distributions near the 5-fold axes. The white arrow in the inset of panel F indicates a position of high electron density formed by putative ions. A corresponding density is not present in the virion structure, as indicated by a black arrow in the inset of panel E. Bars, 50 Å.

**FIG 3:**
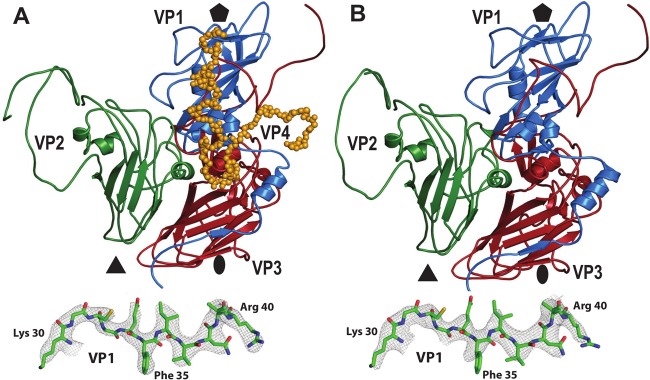
Comparison of icosahedral asymmetric units of IAPV virions and empty particles. Icosahedral asymmetric units of an IAPV virion heated to 63°C (A) and an empty particle (B) are shown as viewed from the particle center. VP1, VP2, VP3, and VP4 are shown in blue, green, red, and orange, respectively. The positions of 5-fold, 3-fold, and 2-fold icosahedral symmetry axes are indicated with pentagons, triangles, and ovals, respectively. The insets at bottom show representative electron densities of the IAPV virion and empty particle at resolutions of 3.26 Å and 3.85 Å, respectively. The maps are contoured at 4 σ.

The genome release of nonenveloped viruses requires capsid disassembly or the formation of pores of sufficient size to allow passage of the genome across the capsid. The incubation of IAPV virions at 63°C resulted in a mixed population of full and empty particles (Fig. 1C). Cryo-EM images of the full particles were used to calculate a single-particle reconstruction to a resolution of 3.3 Å (Table 1; Fig. 2B and E). The structure of the full virion could be built except for residues 1 to 10 and 258 to 318 of VP2 (Fig. 3A). The structure of the minor capsid protein VP4 was better resolved than that in the 4.0-Å-resolution crystal structure of the native virus that was described previously (Fig. 2D and E) (21). Residues 13 to 69 of VP4, among the 69 residues in the polypeptide, could be modeled in the cryo-EM map (Fig. 3A). The positioning of residue 69 from the C terminus of VP4 close to residues Asp186-Asp187-Phe188 of VP1 verifies the previous speculation that these residues form a putative catalytic site responsible for the cleavage of dicistrovirus VP0 into VP4 and VP3 subunits (21).

**TABLE 1:** Cryo-EM structure quality indicators for full and empty IAPV particles

Parameter	Value or description for IAPV heated at 63°C	
	Full virions	Empty particles
EMDB/PDB codes	EMD-4114/5LWG	EMD-4115/5LWI
Resolution (Å)	3.26	3.85
*R*_work_	0.314	0.366
No. of atoms^*a*^	6,373	5,876
RMSD		
Bond length (Å)	0.007	0.006
Bond angle (°)	1.28	1.22
Ramachandran region (%)		
Favored^*b*^	89.18	89.02
Allowed^*b*^	10.32	10.44
Outliers^*b*^	0.50	0.54
% poor rotamers^*b*^	0.84	0.45
Clashscore (percentile)^*b*^	3.09 (100)	3.96 (100)
MolProbity score (percentile)	1.67 (100)	1.76 (100)
C-β deviation (%)^*b*^	0.26	0.14
Average atomic B factor	52.2	68.4

a For one icosahedral asymmetric unit.

b According to the criteria of Molprobity (62).

The structure of the heated IAPV virion is nearly identical to that of the native virus (Fig. 2A, B, D, and E), with a root mean square deviation (RMSD) of C-α atoms from the icosahedral asymmetric units of 0.76 Å (Table 2). Accordingly, the internal volumes of the virion cavities are 6.2 × 10^6^ Å^3^ and 6.3 × 10^6^ Å^3^, respectively. This structural similarity indicates that IAPV does not form A particles before genome release. These results are similar to the previous observation that TrV virions do not expand before genome release (24). In contrast, the genome release of enteroviruses is preceded by the formation of A particles that are characterized by a 5% increase in virion diameter and by the formation of channels in the capsid (25, 31–33).

**TABLE 2:** Structural comparison of icosahedral asymmetric units of IAPV in different assembly forms

IAPV form	RMSD for indicated form		
	Pentamer	Empty particle	Heated virion
Native virion	1.09	0.93	0.80
Heated virion	0.87	0.68	
Empty particle	0.87		

### Comparison of RNA distributions in native and heated IAPV virions.

Whereas the capsid structures of the native and heated (63°C) IAPV virions were almost identical, there were differences in the distribution of the genomic RNA inside the particles (Fig. 2). The genome of IAPV is a 9,500-nucleotide single-stranded RNA (ssRNA) molecule which is packed inside the icosahedral capsid (9). The RNA molecule cannot entirely follow the icosahedral symmetry of the capsid. Both the X-ray crystallography and single-particle reconstruction methods used for IAPV structure determination employ icosahedral symmetry to calculate the three-dimensional (3D) electron density maps. Therefore, both of the methods used to determine IAPV structures provide electron density maps that contain information about the icosahedrally symmetrized distribution of the genome. In the native virus, the RNA uniformly fills the virion cavity in an approximate sphere with a radius of 110 Å (Fig. 2D). Although direct interactions of the RNA with capsid proteins were not observed, it might be possible that the genome interacts with N-terminal parts of the capsid proteins VP1 and VP2 that are not resolved in the virion structure, as previously speculated for picornaviruses (28, 39). In parechoviruses of the family Picornaviridae, portions of the RNA interact specifically with the capsid and are therefore resolved in electron density maps calculated with icosahedral symmetry (40–42). However, the current structures show that the IAPV genome is folded in the capsid cavity, without any icosahedral ordering imposed by the surrounding capsid proteins (Fig. 2D and E).

In heated IAPV virions, the RNA forms a 20-Å-thick shell with a higher density that tightly follows the inner face of the capsid and a sphere in the center of the virion with a radius of 80 Å (Fig. 2E). These two volumes are separated by a spherical shell of lower RNA density with a diameter of 80 to 90 Å. The genome in the heated IAPV virions did not form specific contacts with the capsid, similar to what was previously shown for A particles of enteroviruses (29, 34). The changes in the distribution of RNA may facilitate the subsequent release of the genome from the IAPV virion.

### Genome release of IAPV is connected to detachment of VP4 subunits from the capsid.

**FIG 4:**
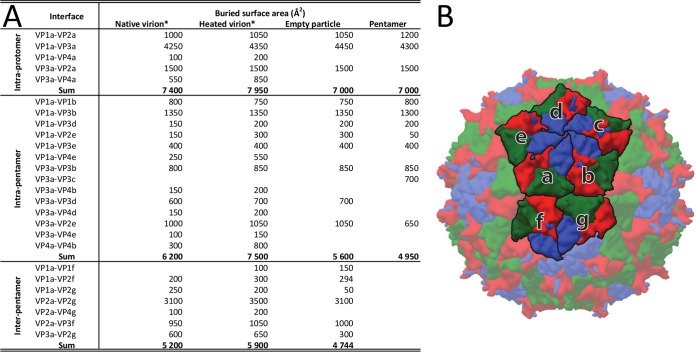
Buried surface areas of interfaces within IAPV virions at different temperatures, within empty particles, and within pentamers. (A) Buried surface areas. Individual subunits are labeled according to their relative positions as shown in panel B. (B) Capsid surface representation of an IAPV virion, with VP1, VP2, and VP3 subunits shown in blue, green, and red, respectively. Icosahedral asymmetric units considered for buried surface calculations are labeled with letters. The buried surface areas were calculated using the PISA server (61). *, note that the differences in buried surface areas between the native and heated virions are due to the extra residues and side chains of VP4 that could be built into the heated virion structure. The differences do not correspond to major conformational changes.

**FIG 5:**
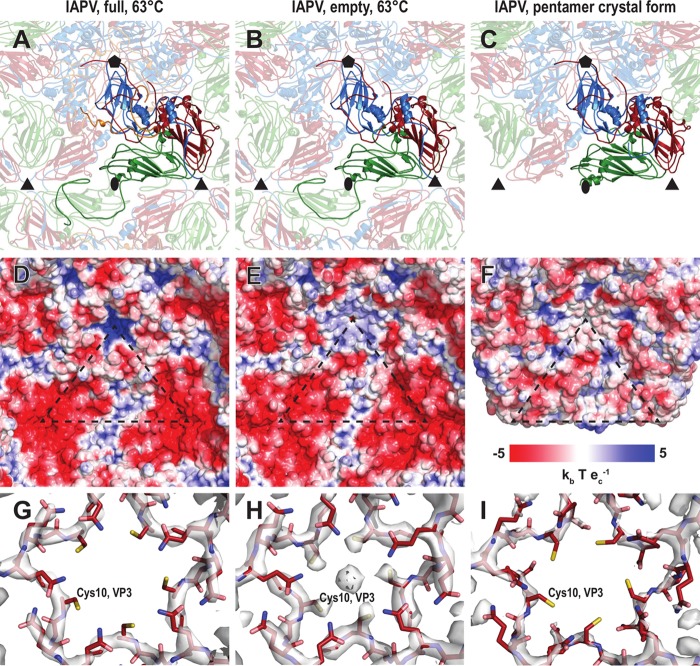
Comparison of intersubunit interactions and charge distributions in IAPV virions, empty particles, and pentamers. Cartoon representations of an IAPV virion heated to 63°C (A), an empty particle (B), and a pentamer (C) are shown as viewed from the particle center. VP1 subunits are shown in blue, VP2 in green, VP3 in red, and VP4 in orange. Selected subunits are shown in bright colors. The positions of 5-fold, 3-fold, and 2-fold icosahedral symmetry axes are indicated with pentagons, triangles, and ovals, respectively. (D to F) Molecular surface of the capsid interior, colored according to the charge distribution. (G to I) Distributions of electron density close to the 5-fold axis. The maps are contoured at 4 σ.

Genome release from IAPV virions results in the formation of empty capsids that are the same size as native virions (Fig. 2). The structure of the empty capsid could be built except for residues 1 to 21 and 258 to 318 of VP2 (Fig. 3B). There were no changes in the positions of subunits VP1 to VP3 relative to those in the native virus, and the structures of the icosahedral asymmetric units had a C-α atom RMSD of 0.98 Å. However, whole VP4 subunits and residues 1 to 21 of the N terminus of VP2 were not resolved for the empty capsids (Fig. 3B). As a consequence, the volume of the capsid cavity increased to 7.0 × 10^6^ Å^3^. Furthermore, the loss of the structure of the VP2 N terminus resulted in a reduction in intrapentamer interfaces, from 5,900 Å^2^ to 5,150 Å^2^ (Fig. 4). It is not likely that this limited reduction of protein contacts causes a biologically important reduction in capsid stability. However, the loss of the structure of the VP2 N terminus and of VP4 (Fig. 5A and B) resulted in changes of the distribution of charges inside the capsids (Fig. 5D and E). It is interesting that the empty capsid does not contain any pores that might serve for the release of the genome and the VP4 subunits. It is possible that the pores are transitory and close after the genome and VP4 subunits are released. Furthermore, the imposition of icosahedral symmetry during calculations for cryo-EM reconstruction may obscure a unique pore found in the capsid. In contrast, the empty capsids produced after enterovirus genome release, which were named B particles, are similar in structure to A particles and contain two types of pores in their capsid walls (29).

The previously determined structure of the TrV virion lacked a resolved electron density for VP4 subunits (22). However, it was shown that TrV virions contain VP4 peptides, and dissolved TrV crystals could be used to infect triatoma beetles (22–24). It was therefore speculated that VP4 peptides are unstructured components of TrV virions. In contrast, electron density maps enabled the building of the structure of VP4 in cricket paralysis virus (CrPV) (19). It was proposed that viruses closely related to TrV might form a separate genus within the family Dicistroviridae, characterized by the absence of structured VP4 subunits (22). Our results show that IAPV virions and empty particles are distinguished by the presence of structured VP4 subunits and 11 residues from the N terminus of VP2.

### Putative mechanism ensuring the inclusion of VP4 in dicistrovirus virions.

It was shown previously that empty TrV capsids disassemble into pentamers of capsid protein protomers composed of VP1, VP2, and VP3 that subsequently assemble into lip-shaped particles formed by two pentamers of capsid protein protomers facing each other with their bases (24). Our previous crystallographic analysis of IAPV identified conditions that gave rise to crystals containing a similar lip-shaped assembly of IAPV capsid proteins (21). Furthermore, lip-shaped particles also copurified with native IAPV virions (Fig. 1B). The crystallized IAPV lip-shaped particles lacked a resolved density for VP4 and residues 1 to 58 of the N terminus of VP2 (Fig. 5C). These changes in the pentamer structure resulted in removal of the strong negative charge that was distributed in areas around the 3-fold icosahedral symmetry axes in the full and empty IAPV virions (Fig. 5D to F). The overall structure of the protomer remained very similar to the one observed for the native virus (Table 2). The tendency of capsid proteins to form these lip-shaped particles may interfere with capsid formation. However, it was speculated that during virus assembly, the formation of the lip-shaped particles is prevented by the presence of VP4 residues that are at the time part of VP0 (24). It is unlikely to be a coincidence that the capsid proteins of two different viruses that share only 22% sequence identity are capable of forming similar lip-shaped particles unless it boosts virus fitness. We therefore speculate that the formation of the lip-shaped particles may be a mechanism preventing the assembly of aberrant capsids lacking VP4. It was speculated previously that the cleavage of VP0 into VP4 and VP3 is catalyzed by a DDF sequence that is exposed on the inside of the capsid. Such cleavage might occur in pentamers, and it is possible that the VP4 peptide would then dissociate from the complex. The resulting aberrant pentamers might be incorporated into virions that would then lack VP4 subunits. However, if the VP4-lacking pentamers formed lip-shaped particles, then they could not interfere with virus assembly.

### Cations positioned inside empty IAPV particles at 5-fold axes.

Cryo-EM reconstruction of the empty IAPV capsid shows a strong electron density located on 5-fold axes close to the inner surface of the capsid, whereas the volume is occupied by VP4 subunits in the virions (Fig. 2D to F). The intensity of this density is similar to that of the surrounding capsid proteins (Fig. 2E); however, it becomes weaker and less resolved after B-factor sharpening (Fig. 5H). The density is located in the vicinity of side chains of five symmetry-related Cys10 residues of VP3 subunits (Fig. 5H). Similar density could not be observed in the full virions (Fig. 5G). The inner surface of the capsid is positively charged in the virions, whereas due to the conformational changes of the VP1 and VP4 subunits, there are negatively charged pockets at the 5-fold axes in the empty IAPV capsid (Fig. 5D and E). We therefore speculate that the extra density belongs to positively charged ions, such as Ca^2+^ or Mg^2+^. The side chains of the five cysteines do not provide optimal coordination for cations that are not positioned exactly on the 5-fold axis (Fig. 5H). The icosahedrally averaged map therefore contains a somewhat smeared density of the ions (Fig. 2E). The density of the putative cations was not observed in the pentamers (Fig. 5I).

### Comparison to genome release and delivery of TrV and enteroviruses.

Current knowledge of the genome release processes of small nonenveloped viruses, particularly those of the order Picornavirales, is based predominantly on structural analyses of conformational changes of their capsids before and after genome release. Most studies have focused on human enteroviruses, since the genus includes important human pathogens, such as poliovirus, rhinoviruses, and enterovirus 71 (EV71) (36, 43–45). Pores positioned around the 5-fold and 2-fold axes of the icosahedral symmetry of the capsids were speculated to be the channels for genome release (46, 47). Nevertheless, the observed pores were never of a sufficient size to allow passage of the genome, and additional structural changes to the capsid would be required for genome release. Furthermore, a study of an asymmetric interaction of coxsackievirus B3 with a receptor inserted into a nanodisc showed limited particle expansion and indicated that the genome might be released through a specific pore formed along the 2-fold or 3-fold axis of symmetry of the capsid (48). Previous studies of dicistrovirus genome release were limited to the analysis of empty TrV particles at a resolution of 20 Å (24).

It was previously indicated that the genome release of TrV was connected to changes in the orientations of capsid proteins that were proposed to move as rigid blocks in so-called “tectonic movements” in the RMSD ranges of 2.5 to 11.4 Å (22, 24). Furthermore, it was postulated that a partial capsid cracking or disassembly is required for TrV RNA externalization because there are no obvious pores for RNA egress and empty TrV capsids disassemble upon or after genome release (24). In contrast, the structures and positions of the major capsid proteins VP1, VP2, and VP3 in IAPV virions and empty particles are almost identical (Fig. 3). Furthermore, the structures of empty TrV particles were interpreted as containing narrower channels along 5-fold axes of icosahedral symmetry than is the case in the native virus (24). However, IAPV particles do not contain holes in the capsids, irrespective of whether they are empty or full (Fig. 2 and 5).

The reconstructions of full and empty TrV capsids differed in a “blob” of electron density located on the outside of the capsid, on the 2-fold axes (24). This density was interpreted as a trace of the RNA genome in the process of release from the particle (24). Therefore, a putative channel located at the 2-fold axis of the capsid was speculated to be the place for TrV genome egress. This indicated that TrV genome release is similar to that of enteroviruses (24). The formation of enterovirus A particles is characterized by movements of α3 helices from VP2 subunits away from the icosahedral 2-fold axis, which results in the formation of a 10- by 5-Å pore that was speculated to be utilized for genome release (36, 43–45). However, empty IAPV particles have compact capsids, and the α3 helices remain in the same position in the empty capsid as in the native virus (Fig. 5A and B). There is no evidence that the area around the 2-fold axis should serve as a channel for IAPV genome release. However, it is possible that asymmetric rearrangements of IAPV capsids were averaged out in the icosahedral reconstruction or that short-lived capsid conformations with pores could not be captured by the use of heat to trigger genome release.

Enterovirus A particles contain pores in their capsids, have N termini of VP1 subunits exposed at the virion surface, and release VP4 subunits (46, 47). Residues from N-terminal regions of VP1 subunits were shown to form amphipathic helices, which disrupt endosomal membranes and together with VP4 subunits allow the transport of enterovirus genomes to the cell cytoplasm (31, 49, 50). For the native virions of IAPV, all the residues from the N terminus of VP1 are resolved in the electron density maps. Furthermore, the structure of the N terminus of VP1 of IAPV remains the same in the empty capsid (Fig. 3A and B and 5A and B). Thus, the N terminus of VP1 of IAPV cannot interact with a lipid bilayer. Moreover, a pore at the base of the canyon, which was shown to be the site of externalization of VP1 subunits for coxsackievirus 16 (29, 36), is not present in the empty particles of IAPV. The compact structure of empty IAPV particles together with the previously published negative results for IAPV pentamer-induced liposome lysis (21) indicates that the N termini of IAPV VP1 subunits are unlikely to interact with membranes. The mechanism by which IAPV delivers its genome across the biological membrane requires further study.

The structures of heat-treated full and empty particles indicate that the RNA release mechanism of IAPV is different from that of enteroviruses, such as coxsackievirus 16, human rhinovirus 2, and poliovirus 1 (36, 43–45). Dicistroviruses do not form A particles, and the genome is probably released through a transiently formed pore in the capsid wall that closes after genome egress.

## MATERIALS AND METHODS

### Virus propagation.

The propagation of IAPV in honeybee pupae and subsequent purification were carried out as described previously (21).

### Preparation of IAPV A particles by heating.

Virions at a concentration of 0.02 mg/ml in 0.25 M HEPES, pH 7.5, 0.25 M NaCl buffer were incubated with Sybr green II (diluted 3,000 times from the stock solution according to the manufacturer's instructions), and the mixture was heated from 25 to 95°C in 1°C increments, with a 2-min incubation time at each temperature, in a real-time PCR instrument (Roche LightCycler 480). The fluorescence signal increases as the dye interacts with RNA that is released from thermally destabilized particles, or the dye might be able to enter the particles. The thermal stability of the virus was estimated as the temperature corresponding to an increase in fluorescence to 50% of the maximal value obtained when all virions were thermally denatured. The measurements were carried out in triplicate.

### Cryo-electron microscopy of IAPV particles.

A solution of freshly purified IAPV (3.5 μl at 2 mg/ml) was heated to 63°C for 10 min in a thermocycler and stored on ice until it was applied to holey carbon grids (Quantifoil R2/1, 300 mesh; Quantifoil Micro Tools) and plunge frozen using an FEI Vitrobot Mark IV machine, set to a 2-s blotting time and a −2 blot force. The Vitrobot sample application chamber was held at 25°C and 100% humidity during the whole vitrification process. Grids with the vitrified sample were transferred to an FEI Titan Krios electron microscope operated at 300 kV and aligned for parallel illumination in nanoprobe mode. Images were recorded with an FEI Falcon II direct electron detection camera under low-dose conditions (20 e^−^/Å^2^), with underfocus values ranging from 1.0 to 3.0 μm at a nominal magnification of ×75,000, resulting in a pixel size of 1.07 Å/pixel. Each image was recorded in movie mode with 0.5 s of total acquisition time and saved as seven separate movie frames. In total, 1,381 micrographs were acquired.

### Icosahedral reconstruction of full and empty particles of IAPV.

The reconstructions of full and empty IAPV particles were calculated independently according to the pipeline described below. The movie frames from each exposure were aligned to compensate for drift and beam-induced motion during image acquisition by using the program SPIDER (51). Regions with IAPV particles (450 × 450 pixels) were manually picked and extracted from the micrographs by using the program e2boxer.py from the package EMAN2 (52), resulting in 2,386 empty-particle and 25,176 full-particle images. Subsequently, the particles were separated into two half-data sets for all of the subsequent reconstruction steps to follow the “gold standard” procedure for resolution determination (53). The contrast transfer function (CTF) parameters of each micrograph were automatically estimated using the program ctffind4 (54). The images were processed using the package RELION 1.4 (55). The particles were subjected to multiple rounds of two-dimensional (2D) classification and 3D classification, resulting in a nearly homogeneous set of IAPV particles. A low-pass-filtered (60 Å) structure of the native IAPV virion determined by X-ray crystallography was used as an initial model for 3D classification and for subsequent refinement, which was performed using the RELION 3dautorefine procedure. To further homogenize the data set, particles were subjected to another round of 3D classification, omitting the alignment step and using the particle shifts and orientations estimated in the previous refinement step. This classification assumes that the shifts and orientations of the particles were accurately estimated during the refinement step and that the particles are distributed solely among the classes depending on their unique features. This usually helps to discard particles that do not contribute high-resolution information to the particle reconstruction. Final reconstruction, performed according to the gold standard (53), was calculated with RELION 3dautorefine (55). The resulting map was masked with a threshold mask and B-factor sharpened using the RELION postprocess procedure (56). The B factors applied for sharpening were −102.9 Å^2^ and −107.1 Å^2^ for the full- and empty-particle IAPV reconstructions, respectively. The resulting resolutions were estimated to be 3.26 and 3.85 Å for the full and empty IAPV particles, respectively, at the 0.143 Fourier shell correlation (FSC) cutoff.

### Cryo-EM structure determination and refinement.

The initial model, derived from the native IAPV virion structure (19), was fitted into the B-factor-sharpened cryo-EM map and subjected to manual rebuilding using the programs Coot and O and to coordinate and B-factor refinement using the programs Refmac5 and Phenix (57–60).

### Accession number(s).

Cryo-EM electron density maps of the IAPV virion and empty particle were deposited with the Electron Microscopy Data Bank (EMDB) under accession numbers EMD-4114 and EMD-4115, respectively, and the fitted coordinates were deposited in the Protein Data Bank (PDB) under accession codes 5LWG and 5LWI, respectively.
